# Attitudes and Preferences Regarding the Use of Rapid Self-Testing for Sexually Transmitted Infections and HIV in San Diego Area Men Who Have Sex With Men

**DOI:** 10.1093/ofid/ofz043

**Published:** 2019-03-11

**Authors:** Teresa A Cushman, Susannah K Graves, Susan J Little

**Affiliations:** 1 Division of Infectious Diseases, University of Colorado Denver, Aurora, Colorado; 2 Division of Infectious Diseases, University of California San Diego, San Diego, California

**Keywords:** HIV, HIV testing, men who have sex with men (MSM), point of care, sexually transmitted infection (STI), STI testing

## Abstract

**Background:**

Sexually transmitted infections (STIs) increase the risk of HIV transmission and are present at high rates among men who have sex with men (MSM). Adherence to HIV/STI testing guidelines is low in the United States. Testing programs that utilize rapid self-administered HIV/STI tests improve testing rates, though multiple factors influence their uptake.

**Methods:**

MSM were recruited at an HIV/STI testing and treatment program in 2014 and provided consent, demographics, risk behaviors, HIV/STI test preferences, and perceived testing barriers via an online questionnaire. Comparisons of testing preferences and barriers were made based on age, risk group, and HIV serostatus using the Fisher exact test.

**Results:**

HIV testing preferences included rapid oral test (71.1%), home test location (78.5%), electronic delivery of HIV-negative test results (76.4%), and direct provider notification for HIV-positive test results (70%), with respondents age >45 years being significantly more likely to prefer home testing (*P* = .033). STI testing preferences included self-collection of specimens (73.2%), home test location (61%), electronic delivery of negative STI test results (76.4%), and direct provider notification for positive STI test results (56.6%) with no significant differences between age, HIV serostatus, or risk groups. The most frequently reported HIV and STI testing barrier was lack of known prior HIV/STI exposure (57.3% for HIV, 62.9% for STI) with respondents age <45 years more frequently citing inconvenience as a barrier to testing (HIV: 50.9% vs 17.4%, *P* = .010; STI: 58.3% vs 31.8%, *P* = .070).

**Conclusions:**

Although additional research is needed, increasing resources directed specifically toward home testing has the potential to translate into improved uptake of rapid HIV/STI testing. Efforts to improve convenience in testing programs must be balanced with the need for continued educational outreach.

HIV and bacterial sexually transmitted infections (STIs), including syphilis, *Neisseria gonorrhea* (NG), and *Chlamydia trachomatis* (CT), remain a significant public health problem in the United States, and men who have sex with men (MSM) continue to be disproportionately affected by these infectious diseases [1, 2]. An estimated 15% of individuals living with HIV are unaware of their diagnosis [3]. The diagnosis of an STI is a well-established risk factor for HIV acquisition [4]. In recognition of the key preventive role played by routine testing, the US Centers for Disease Control and Prevention (CDC) recommends at least annual HIV and STI screening in all sexually active MSM, with more frequent testing for individuals who engage in high-risk behavior [5, 6]. Despite these recommendations, rates of testing remain below recommended levels in the United States [7, 8].

A wealth of data supports the feasibility and acceptability of self-administered HIV and STI sample collection and testing [9, 10]. Advances in molecular diagnostics have made rapid self-testing increasingly feasible and affordable, and improved global access to digital technology has allowed for improved access to testing services. Utilizing these advances, novel HIV/STI testing programs promoting the use of rapid self-testing outside the traditional clinic setting have shown increased uptake of testing, decreased time to testing, and increased diagnosis of early infection [11–15]. However, other studies have failed to demonstrate these benefits [16]. Considerable heterogeneity exists between the structural elements of these programs, including the type of rapid HIV test utilized (oral, dried blood spot, minitube) [11, 15, 16], the venue at which HIV/STI rapid self-testing is available for use (walk-in clinic, home) [13, 14], and test result delivery (electronically via secure website, provider phone call, text message) [11, 12, 15], which may explain the differences seen in program uptake. Although the CDC now supports the incorporation of HIV self-testing into large-scale testing programs [17], further investigation of the optimal utilization of the elements of self-testing programs is needed.

## METHODS

We performed a cross-sectional study of a convenience sample of MSM in San Diego, California, to improve our understanding of prevailing attitudes and preferences regarding commercially available rapid HIV and 3-site GC/CT testing methods and perceived barriers to routine HIV/STI testing in order to optimize an existing community-based testing program targeting MSM and guide future program expansion efforts to promote uptake among high-risk individuals. Men at least 18 years of age who were sexually active in the past 3 months with at least 1 male sexual partner were invited to complete an anonymous online survey (SurveyMonkey) at a community-based HIV/STI screening program from October through December of 2014 via an advertising banner on their electronic and paper HIV/STI test result sheets. Upon completion of the survey, respondents received a link to a $5 electronic gift card. Respondents were not required to answer all questions in order to complete the survey. Participation was anonymous, and no personal identifying information was collected. Survey questions collecting demographic information, risk behaviors in the past 12 months, HIV/STI testing history, and barriers to HIV/STI testing were adapted from a previously published CDC HIV testing perspectives survey tool [18]. Respondents were asked to select any of the listed reasons that had ever prevented them from testing, and multiple responses were allowed. Respondents were categorized as engaging in high-risk behavior if they reported 2 or more of the following behaviors: unprotected anal intercourse in the last 3 months, more than 5 sex partners in the past 12 months, sex in exchange for money or drugs in the past 12 months, self-reported STI diagnosis or sexual activity under the influence of recreational drugs/alcohol in the past 12 months. Respondents who reported that they had engaged in none or only 1 of these behaviors were categorized as low risk. Respondents were then asked to give their opinion regarding multiple components of a hypothetical HIV/STI testing program. Questions pertaining to HIV and STI were asked separately. Only HIV-negative respondents were asked to provide HIV testing preferences. Respondents were instructed to assume that the services in this hypothetical testing program would be free of charge. The hypothetical testing program components included commercially available tests for HIV (rapid oral swab, rapid dried blood spot, traditional blood draw) and STI (self- or provider-collected urine and oral/rectal swabs for NG/CT), locations where HIV/STI testing could be obtained and performed (home, traditional clinic setting, community outreach event or mobile testing van, electronic kiosk at a public location such as a bar or bathhouse), and test result delivery methods (login to a secure website, unsecure e-mail or text message, health provider phone call or clinic visit, letter in the mail). Respondents were then asked to directly rank their preferred test type, venue, and result delivery method. The study protocol and all study-related procedures were approved by the UCSD Human Research Protections Program, including a waiver of consent.

The primary outcome was test venue preference. Secondary outcomes included test type preference, specimen collection technique preference, test result delivery preferences, perceived barriers to testing, and appropriateness of testing interval based on risk group, as defined by the current CDC guidelines for HIV and STI testing for sexually active MSM [5, 6]. Primary and secondary outcomes were compared between HIV serostatus, age, and risk groups using the Fisher exact test at an alpha level of 0.05. Statistical analysis was performed with SPSS, version 24 (SPSS Inc., Chicago, IL).

## RESULTS

**Figure 1.**
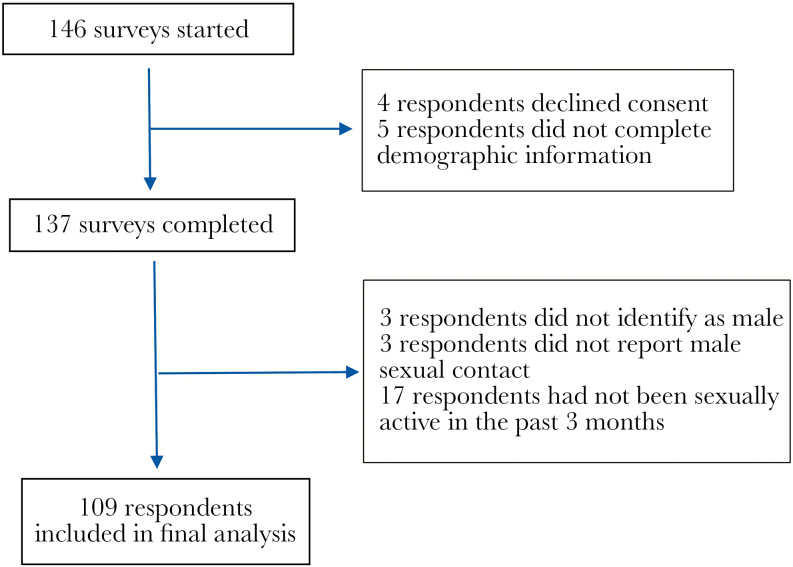
Survey response and exclusion.

Of the 137 surveys completed, 28 were excluded (Figure 1). The baseline characteristics of the 109 MSM included in the final analysis are shown in Table 1. Seventy-six respondents (69.7%) were classified as high risk. Eighty-eight respondents (80.7%) reported a negative HIV serostatus at the time of most recent testing and were included in the analysis of HIV testing preferences. All respondents who provided responses were included in the analysis of STI testing preferences and perceived HIV/STI testing barriers.

**Table 1.** Demographic and Behavioral Risk Characteristics

Characteristic	All Participants (n = 109), No. (%)
Race	
White	81 (74.3)
Black	8 (7.3)
Hispanic	14 (12.8)
Other	6 (5.5)
Age	
≤45 y	76 (69.7)
≥46 y	33 (30.3)
Education	
GED/HS diploma only	82 (75.2)
Bachelor’s degree or higher	27 (24.8)
Income	
<$20 000	15 (13.8)
$20 000–$60 000	39 (35.8)
>$60 000	55 (50.5)
Risk group	
MSM	109 (100)
MSM + IDU	3 (2.8)
Risk behaviors	
Unprotected sex	92 (84.4)
>5 partners in past 12 mo	70 (64.2)
Sex work	4 (3.7)
Self-reported STI in past 12 mo	33 (30.3)
Use of drugs or alcohol during sex	36 (33.0)
None (none of the above risk factors)	28 (25.7)
Overall risk behavior profile	
High risk (any 2 of the above risk behaviors)	76 (69.7)

Abbreviations: GED, General Education Development certificate; HS, high school; IDU, injection drug user; MSM, men who have sex with men; STI, sexually transmitted infection.

Preference for HIV test type, test location, and result delivery are presented in Table 2. The preferred test type was a rapid oral HIV test (71.1%), and the preferred venue for use of rapid HIV testing was home (78.5%). Respondents preferred to receive negative HIV test results electronically via secure website or text/e-mail (76.4%). However, respondents preferred to receive positive HIV test results directly from a provider, either by phone or an in-person clinic visit (70%). Nearly all respondents (94%) indicated that they would be comfortable making their own follow-up arrangements upon receipt of a positive result outside of a clinic setting. There were no significant differences in HIV testing preferences between age groups or risk behavior groups.

**Table 2.** HIV and STI Testing and Result Notification Preferences

Preference	All Participants, No. (%)	Age ≤45 y, No. (%)	Age ≥46 y, No. (%)	*P* Value
HIV test type	n = 83	n = 64	n = 19	
Rapid oral	59 (71.1)	45 (70.3)	14 (73.7)	1.00
Rapid finger stick	7 (8.4)	7 (10.9)	0 (0)	.34
Traditional clinic test	17 (20.5)	12 (18.8)	5 (26.3)	.52
Rapid HIV test location	n = 79	n = 59	n = 20	
Home	62 (78.5)	47 (79.7)	15 (75.0)	.76
Clinic	12 (15.2)	9 (15.3)	3 (15.0)	1.00
Community health	5 (6.3)	3 (5.1)	2 (10.0)	.60
Retail	0 (0)	0 (0)	0 (0)	n/a
Social venue	0 (0)	0 (0)	0 (0)	n/a
HIV test result notification: negative result	n = 72	n = 53	n = 19	
Electronic: secure website login	24 (33.3)	17 (32.1)	7 (36.8)	.78
Electronic: e-mail or text message	31 (43.1)	22 (41.5)	9 (47.4)	.79
Health provider call or office visit	17 (23.6)	14 (26.4)	3 (15.8)	.53
US mail	0 (0)	0 (0)	0 (0)	n/a
HIV test result notification: positive result	n = 72	n = 53	n = 19	
Electronic: secure website login	10 (13.9)	7 (13.2)	3 (15.8)	.72
Electronic: e-mail or text message	10 (13.9)	9 (17.0)	1 (5.3)	.27
Health provider call or office visit	52 (72.2)	37 (69.8)	15 (78.9)	.56
US mail	0 (0)	0 (0)	0 (0)	n/a
Rapid STI test collection method/location	n = 82	n = 55	n = 27	
Collect own samples at home	50 (61.0)	29 (52.7)	21 (77.8)	.03*
Collect own samples at clinic	10 (12.2)	8 (14.5)	2 (7.4)	.49
Health professional collect in clinic	22 (26.8)	18 (32.7)	4 (14.8)	.11
STI test result notification: negative result	n = 76	n = 51	n = 25	
Electronic: secure website login	23 (30.3)	15 (29.4)	8 (32.0)	1.00
Electronic: e-mail or text message	35 (46.1)	23 (45.1)	12 (48.0)	1.00
Health provider call or office visit	18 (23.7)	13 (25.5)	5 (20.0)	.78
US mail	0 (0)	0 (0)	0 (0)	n/a
STI test result notification: positive result	n = 76	n = 51	n = 25	
Electronic: secure website login	13 (17.1)	7 (13.7)	6 (24.0)	.33
Electronic: e-mail or text message	19 (25.0)	12 (23.5)	7 (28.0)	.78
Health provider call or office visit	43 (56.6)	32 (62.7)	11 (44.0)	.15
US mail	1 (1.3)	0 (0)	1 (4.0)	.33

Abbreviation: STI, sexually transmitted infection.

*P < .05.

Preference for STI test type, test location, and result delivery are presented in Table 2. The preferred test type was self-collection (73.2%), and the preferred venue for specimen self-collection was home (61%). Respondents age >45 years were more likely to prefer home STI specimen collection (77.8% vs 52.7%, *P* = .033). Respondents preferred to receive negative STI test results electronically via secure website or text/e-mail (76.4%). However, respondents preferred to receive positive STI test results directly from a provider, either by phone or an in-person clinic visit (56.6%). Nearly all respondents (97%) indicated that they would be comfortable making their own follow-up arrangements upon receipt of a positive result outside of a clinic setting. There were no significant differences in STI testing and results delivery preferences between serostatus or risk behavior groups.

Perceived barriers to HIV testing are presented in Table 3. The most frequently reported reasons for not receiving HIV testing were a lack of known prior HIV exposure (57.3%), fear/anxiety (45.3%), and inconvenience (40.0%). Respondents age <45 years were significantly more likely to report inconvenience as a barrier to testing (50.9% vs 17.4%, *P* = .010). HIV-positive respondents were less likely to report lack of known prior HIV exposure than HIV-negative respondents (25.0% vs 62.9%, *P* = .028). There were no differences between risk behavior groups.

**Table 3.** Reported Barriers to HIV and STI Testing

Testing Barriers	All Participants, No. (%)	Age Category			Risk Category			HIV Serostatus		
		Age ≤45 y, No. (%)	Age ≥46 y, No. (%)	*P* Value	Lower-Risk Behavior, No. (%)	High-Risk Behavior, No. (%)	*P* Value	HIV-, No. (%)	HIV+, No. (%)	*P* Value
HIV testing barriers	n = 75	n = 52	n = 23		n = 25	n = 50		n = 62	n = 12	
No exposure	43 (57.3)	26 (50.0)	17 (73.9)	.08	16 (64.0)	27 (54.0)	.47	39 (62.9)	3 (25.0)	.02*
Fear/anxiety	34 (45.3)	25 (48.1)	9 (39.1)	.62	8 (32.0)	26 (52.0)	.14	25 (40.3)	9 (75.0)	.05
Privacy concerns	8 (10.7)	6 (11.5)	2 (8.7)	1.00	2 (8.3)	6 (12.0)	.71	8 (12.9)	0 (0)	.34
Fear of needles	2 (2.7)	2 (3.8)	0 (0)	1.00	1 (4.0)	1 (2.0)	1.00	2 (3.2)	0 (0)	1.00
Lack of venue	7 (9.3)	6 (11.5)	1 (4.3)	.43	2 (8.0)	5 (10.0)	1.00	6 (9.7)	0 (0)	.58
Cost	2 (2.7)	2 (3.8)	0 (0)	1.00	1 (4.0)	1 (2.0)	1.00	2 (3.2)	0 (0)	1.00
Inconvenience	30 (40.0)	27 (50.9)	4 (17.4)	.01*	8 (32.0)	22 (44.0)	.45	26 (41.9)	3 (25)	.35
STI testing barriers	n = 70	n = 48	n = 22		n = 19	n = 51		n = 57	n = 13	
No exposure	44 (62.9)	28 (58.3)	16(72.7)	.30	16 (84.2)	28 (54.9)	.03*	39 (68.4)	5 (38.5)	.06
No symptoms	41 (58.6)	28 (58.3)	13 (59.1)	1.00	8 (42.1)	33 (64.7)	.11	33 (57.9)	8 (61.5)	1.00
Fear/anxiety	15 (21.4)	13 (27.1)	2 (9.1)	.12	2 (10.5)	13 (25.5)	.21	11 (19.3)	4 (30.8)	.46
Privacy concerns	11 (15.7)	6 (12.5)	5 (22.7)	.30	2 (10.5)	9 (17.6)	.72	6 (10.5)	5 (38.5)	.03*
Fear of needles	3 (4.3)	2 (4.2)	1 (4.5)	1.00	1 (5.3)	2 (3.9)	1.00	3 (5.3)	0 (0)	1.00
No venue	7 (10.0)	6 (12.5)	1 (4.5)	.42	0 (0)	7 (13.7)	.18	7 (12.3)	0 (0)	.33
Cost	3 (4.3)	3 (6.3)	0 (0)	.55	1 (5.3)	2 (3.9)	1.00	3 (5.3)	0 (0)	1.00
Inconvenience	35 (50.0)	28 (58.3)	7 (31.8)	.07	6 (31.6)	29 (56.9)	.11	28 (49.1)	7 (53.8)	1.00

Participants were asked to choose all that applied; percentages do not sum to 100.

Abbreviation: STI, sexually transmitted infection.

*P < .05.

Perceived barriers to STI testing are presented in Table 3. The most frequently reported reasons for not receiving STI testing were lack of known prior STI exposure (62.9%), absence of STI symptoms (58.6%), and inconvenience (50%). Respondents age <45 years more frequently cited inconvenience as a barrier to testing, though this difference did not meet statistical significance (58.3% vs 31.8%, *P* = .070). Respondents engaged in high-risk behavior were significantly less likely to report lack of known prior STI exposure than those with lower-risk behavior (54.9% vs 84.2%, *P* = .028). HIV-positive respondents were more likely to report privacy concerns as a barrier to STI testing than HIV-negative respondents (30.8% vs 10.5%, *P* = .025).

An evaluation of HIV testing history among HIV-negative respondents is presented in Table 4. Sixty-five respondents reported prior HIV testing within the past 3 months (73%), with an additional 16 respondents reporting prior HIV testing within the past year (18%). High-risk respondents were significantly more likely to have reported testing in the past 3 months (85.0% vs 53.8%, *P* = .001). Lower-risk respondents were significantly more likely to have reported testing in the past 4–12 months (42.3% vs 8.3%, *P* < .001).

**Table 4.** HIV Testing History

Testing History	All HIV-, No. (%)	Lower-Risk Behavior	High-Risk Behavior	*P* Value
Prior HIV test	n = 88	n = 27	n = 61	.30
Yes	87 (98.9)	26 (96.3)	61 (100)	
No	1 (1.1)	1 (3.7)	0 (0)	
Most recent HIV test	n = 86	n = 26	n = 60	
Tested >12 mo ago	5 (5.6)	1 (3.8)	4 (6.5)	1.00
Tested 4–12 mo ago	16 (18.0)	11 (42.3)	5 (8.3)	<.001*
Tested 1–3 mo ago	65 (73)	14 (53.8)	51 (85.0)	.005*

*P < .05.

## DISCUSSION

Respondents expressed a strong preference for the use of a rapid oral test for HIV testing and self-collection of specimens for STI testing in the home. Given the wide variety of feasible alternative HIV testing venues that have been described, including community outreach events/mobile testing programs and kiosks located in public spaces (bar or bathhouse) [19–22], the major strength of this study was the ability of respondents to directly rank these options against the option of home testing. A single study demonstrating a preference for HIV home testing among MSM allowed respondents to directly rank their preferred testing venue but restricted respondents’ options to home- and clinic-based testing [23]. Yang and colleagues also demonstrated a preference for home HIV testing among Australian MSM when allowed to choose between testing at home, at a community-based organization, and at a clinic, though participants only appear to have been given the option to choose their most preferred venue rather than rank all options [24]. As there are currently no commercially available rapid home STI tests in the United States, the only hypothetical alternative STI testing venue described in this survey was the home. A single study demonstrating a preference for home STI testing among MSM also limited testing venue options to home- and clinic-based testing but did not present the option of specimen self-collection in the clinic [25]. A further strength of this study is the stratification of preference by age and risk behavior, which was not performed in the aforementioned studies. Interestingly, respondents age <45 years were somewhat less likely to prefer home specimen collection for STI but did not prefer it for HIV testing. As any STI testing specimens collected at home would require some form of specimen transport to a lab, either via mail or physical dropoff, it is conceivable that young respondents who do not yet live independently or have private transportation may have reasonable privacy concerns related to rapid home STI but not HIV testing.

Our study also evaluated perceived barriers that may limit routine HIV and STI testing. Although nearly 70% of respondents reported engaging in high-risk behaviors, a lack of known prior HIV/STI exposure was the most frequently reported reason for not obtaining both routine HIV and STI testing. Despite longstanding and widespread educational efforts, MSM populations frequently report a lower perceived risk of HIV/STI infection than their self-reported behaviors would indicate [26]. However, high-risk respondents were significantly more likely to have reported HIV testing in the past 3 months and significantly less likely to report lack of known prior STI exposure as a barrier to STI testing. Furthermore, HIV-positive individuals were less likely to report lack of known prior HIV exposure as a prior barrier to HIV testing. These findings would seem to indicate a general awareness of risk status in this cohort, despite their reported barriers. Recent studies have demonstrated an association between receiving multiple negative HIV tests and increased high-risk sexual behavior among MSM [27, 28]. Mustanski and colleagues developed the Inventory of Reactions to Testing HIV Negative (IRTHN) to better quantify diverse reactions to testing negative for HIV, and the initial evaluation revealed that individuals who expressed the belief that a negative HIV test was due to either chance (Luck reaction) or immunity from becoming infected with HIV (Invulnerability reaction) were more likely to be engaged in high-risk sexual behavior [29]. In a subsequent validation of this tool, Feinstein and colleagues replicated the findings of Mustanski et al. and found an association between the belief that a negative HIV test result represented the acceptability of condomless sex (Reinforced Risk reaction) and increased high-risk sexual behavior [30]. Significantly, Feinstein and colleagues also found that lower levels of HIV knowledge, motivation to reduce risk behavior, and behavioral skills to engage in preventive behavior were associated with both the Invulnerability and Reinforced Risk reactions [30]. In addition, although it has become increasingly recognized that the majority of STI infections, particularly at extragenital sites in MSM, are asymptomatic [31], and despite the relatively high socioeconomic status and education level in our cohort, more than half of respondents reported a lack of STI symptoms as a reason for not obtaining routine testing. These results highlight the need for further educational outreach initiatives targeting both HIV knowledge and beliefs regarding the significance of HIV test results in the MSM population.

Inconvenience was cited by many respondents, particularly young individuals, as a barrier to both HIV and STI testing, suggesting that currently utilized testing strategies are not perceived as convenient. Prior research has suggested that the perception of convenience influences the decision to test [32, 33]. Recognition of the increasing importance of convenience in modern health care delivery is reflected in the CDC recommendation to integrate universal HIV screening into routine medical care. As qualitative assessments have revealed that self-testing is perceived as a way to avoid multiple time-consuming clinic visits and make testing more accessible [24, 34], the prioritized integration of home rapid HIV and STI testing options into large-scale testing programs has the potential to address this barrier, particularly among young MSM who are at the greatest risk for acquiring HIV and STIs [1, 2].

Result delivery is another aspect of the testing process that affects convenience. Our study respondents expressed a preference for electronic delivery of negative STI and HIV results, which has been reported in prior studies [35, 36]. With growing access worldwide to electronic communication and social media, this preference will likely continue to increase. A quantitative and qualitative analysis of factors influencing the willingness and barriers to self-HIV testing in MSM in the United Kingdom performed by Flowers and colleagues suggested that rapid test use and acceptability correlated with increasing digital literacy [37]. However, it is important to consider the disadvantages of electronic result notification. Electronic result delivery eliminates health care provider contact, which may deprive an individual of the opportunity to obtain timely and accurate education regarding specific disease symptoms, risk behavior reduction, prevention counseling, and partner notification, which is also reflected in the respondents’ preference for direct provider interaction for delivery of a positive HIV or STI test result. Given the previously noted persistence of high-risk behavior and misinformation regarding the significance of lack of STI symptoms in this cohort, large-scale HIV/STI testing programs should seek to design their programs to balance convenience with the need for continued educational outreach efforts.

In contrast to prior studies [38, 39], only a minority of participants reported privacy concerns as a barrier to HIV and STI testing, though this was more frequently cited among HIV-positive respondents. Multiple recent studies have demonstrated that home-based HIV and STI testing is perceived as more private than traditional clinic-based testing [37, 40]. Efforts to decrease the stigma of HIV and STI, advances in HIV treatment, and increasing acceptance and use of social media and other less secure communication platforms have likely contributed to this trend. As our population was comprised of mostly frequent testers currently utilizing a traditional clinic testing venue, the overall high comfort level with current privacy protections seen in this cohort may not be generalizable to other MSM communities, particularly those who are less frequent testers. Investigation of perceptions regarding the impact of the level of privacy of various result delivery mechanisms on the uptake of self-testing programs remains an area for further research.

This study was subject to several limitations. Although we attempted to robustly describe each of the potential options for test type, venue, and result delivery system, the online survey format did not allow respondents to ask clarifying questions, which may have impacted their reported preferences. Self-reported risk behaviors and subsequent risk stratification were also subject to nonresponse bias due to the survey format. The sample size was relatively small, with only 109 respondents completing the demographic information section and fewer respondents completing all sections of the survey. Completion of the survey required Internet access and was time-consuming, typically requiring 30 minutes to complete, which likely limited participation and thus the generalizability of the results. The population reached by this survey was relatively homogenous and may not be reflective of MSM communities in other geographic regions. Finally, the data set was collected in 2014, and behaviors and/or preferences may have changed since then.

## CONCLUSIONS

Rapid HIV and STI tests for home use are preferred over multiple other potential alternative test venues in San Diego MSM. Although additional research is needed to identify the optimal utilization of multiple aspects of a large-scale HIV and STI self-testing program, increasing resources directed specifically toward home testing has the potential to translate into improved uptake of rapid HIV and STI testing, particularly by reducing inconvenience in the testing process. However, efforts to improve convenience in testing programs should be carefully balanced with the need for continued educational outreach efforts.

## References

[1] Centers for Disease Control and Prevention. Sexually Transmitted Disease Surveillance 2016 September, 2017 https://www.cdc.gov/std/stats16/CDC_2016_STDS_Report-for508WebSep21_2017_1644.pdf Accessed 27 May 2018.

[2] Centers for Disease Control and Prevention. HIV Surveillance Report 2016 November, 2017 https://www.cdc.gov/hiv/pdf/library/reports/surveillance/cdc-hiv-surveillance-report-2016-vol-28.pdf Accessed 27 May 2018.

[3] Centers for Disease Control and Prevention. HIV in the United States: at a glance | statistics overview 2017. https://www.cdc.gov/hiv/statistics/overview/ataglance.html Accessed 27 May 2018.

[4] Katz DA, Dombrowski JC, Bell TR, et al. HIV incidence among men who have sex with men after diagnosis with sexually transmitted infections. Sex Transm Dis 2016; 43:249–54. 26967302 10.1097/OLQ.0000000000000423 PMC4789769

[5] Workowski KA, Bolan GA; Centers for Disease Control and Prevention Sexually transmitted diseases treatment guidelines, 2015. MMWR Recomm Rep 2015; 64:1–137. PMC5885289 26042815

[6] Branson BM, Handsfield HH, Lampe MA, et al; Centers for Disease Control and Prevention (CDC) Revised recommendations for HIV testing of adults, adolescents, and pregnant women in health-care settings. MMWR Recomm Rep 2006; 55:1–17; quiz CE1–4. 16988643

[7] Flagg EW, Weinstock HS, Frazier EL, et al. Bacterial sexually transmitted infections among HIV-infected patients in the United States: estimates from the medical monitoring project. Sex Transm Dis 2015; 42:171–9. 25763669 10.1097/OLQ.0000000000000260 PMC6921480

[8] Kwan CK, Rose CE, Brooks JT, et al. HIV testing among men at risk for acquiring HIV infection before and after the 2006 CDC recommendations. Public Health Rep 2016; 131:311–9. 26957666 10.1177/003335491613100215 PMC4765980

[9] Lunny C, Taylor D, Hoang L, et al. Self-collected versus clinician-collected sampling for chlamydia and gonorrhea screening: a systemic review and meta-analysis. PLoS One 2015; 10:e0132776. 26168051 10.1371/journal.pone.0132776 PMC4500554

[10] Conway DP, Guy R, Davies SC, et al; Sydney Rapid HIV Test Study Rapid HIV testing is highly acceptable and preferred among high-risk gay and bisexual men after implementation in sydney sexual health clinics. PLoS One 2015; 10:e0123814. 25898140 10.1371/journal.pone.0123814 PMC4405382

[11] Jamil MS, Prestage G, Fairley CK, et al. Effect of availability of HIV self-testing on HIV testing frequency in gay and bisexual men at high risk of infection (FORTH): a waiting-list randomised controlled trial. Lancet HIV 2017; 4:e241–50. 28219619 10.1016/S2352-3018(17)30023-1

[12] Wilson E, Free C, Morris TP, et al. Internet-accessed sexually transmitted infection (e-STI) testing and results service: a randomised, single-blind, controlled trial. PLoS Med 2017; 14:e1002479. 29281628 10.1371/journal.pmed.1002479 PMC5744909

[13] Fisher M, Wayal S, Smith H, et al; Brighton Home Sampling Kit Project Steering Group Home sampling for sexually transmitted infections and HIV in men who have sex with men: a prospective observational study. PLoS One 2015; 10:e0120810. 25848769 10.1371/journal.pone.0120810 PMC4388635

[14] Whitlock GG, Gibbons DC, Longford N, et al Rapid testing and treatment for sexually transmitted infections improve patient care and yield public health benefits. Int J STD AIDS 2018; 29:474–82. 29059032 10.1177/0956462417736431 PMC5844454

[15] Page M, Atabani SF, Wood M, et al. Dried blood spot and mini-tube blood sample collection kits for postal HIV testing services: a comparative review of successes in a real-world setting. Sex Transm Infect 2019; 95:43–5. 30072393 10.1136/sextrans-2018-053567

[16] Read TR, Hocking JS, Bradshaw CS, et al. Provision of rapid HIV tests within a health service and frequency of HIV testing among men who have sex with men: randomised controlled trial. BMJ 2013; 347:f5086. 24004988 10.1136/bmj.f5086 PMC3762440

[17] Centers for Disease Control and Prevention. Implementing HIV testing in nonclinical settings: a guide for HIV testing providers March 2016. https://www.cdc.gov/hiv/pdf/testing/CDC_HIV_Implementing_HIV_Testing_in_Nonclinical_Settings.pdf Accessed 27 May 2018.

[18] Centers for Disease Control and Prevention. Evaluation toolkit: patient and provider perspectives about routine HIV screening in health care settings March 2012. https://www.cdc.gov/hiv/topics/testing/healthcare/index.htm Accessed 27 May 2018.

[19] Liang TS, Erbelding E, Jacob CA, et al. Rapid HIV testing of clients of a mobile STD/HIV clinic. AIDS Patient Care STDS 2005; 19:253–7. 15857197 10.1089/apc.2005.19.253

[20] Young SD, Daniels J, Chiu CJ, et al. Acceptability of using electronic vending machines to deliver oral rapid HIV self-testing kits: a qualitative study. PLoS One 2014; 9:e103790. 25076208 10.1371/journal.pone.0103790 PMC4116256

[21] Bowles KE, Clark HA, Tai E, et al. Implementing rapid HIV testing in outreach and community settings: results from an advancing HIV prevention demonstration project conducted in seven U.S. cities. Public Health Rep 2008; 123(Suppl 3):78–85. 10.1177/00333549081230S310 PMC2567007 19172705

[22] Stafylis C, Natoli LJ, Murkey JA, et al. Vending machines in commercial sex venues to increase HIV self-testing among men who have sex with men. Mhealth 2018; 4:51. 30505849 10.21037/mhealth.2018.10.03 PMC6232061

[23] Sharma A, Stephenson RB, White D, Sullivan PS Acceptability and intended usage preferences for six HIV testing options among internet-using men who have sex with men. Springerplus 2014; 3:109. 24600551 10.1186/2193-1801-3-109 PMC3942559

[24] Yang M, Prestage G, Maycock B, et al. The acceptability of different HIV testing approaches: cross-sectional study among GMSM in Australia. Sex Transm Infect 2014; 90:592–5. 25015651 10.1136/sextrans-2013-051495

[25] Pearson WS, Kreisel K, Peterman TA, et al. Improving STD service delivery: would American patients and providers use self-tests for gonorrhea and chlamydia? Prev Med 2018; 115:26–30. 30096329 10.1016/j.ypmed.2018.08.007

[26] MacKellar DA, Valleroy LA, Secura GM, et al; Young Men’s Survey Study Group Perceptions of lifetime risk and actual risk for acquiring HIV among young men who have sex with men. AIDS Behav 2007; 11:263–70. 16791527 10.1007/s10461-006-9136-0

[27] Hoenigl M, Anderson CM, Green N, et al. Repeat HIV-testing is associated with an increase in behavioral risk among men who have sex with men: a cohort study. BMC Med 2015; 13:218. 26444673 10.1186/s12916-015-0458-5 PMC4596465

[28] Fernyak SE, Page-Shafer K, Kellogg TA, et al. Risk behaviors and HIV incidence among repeat testers at publicly funded HIV testing sites in San Francisco. J Acquir Immune Defic Syndr 2002; 31:63–70. 12352152 10.1097/00126334-200209010-00009

[29] Mustanski B, Rendina HJ, Greene GJ, et al. Testing negative means I’m lucky, making good choices, or immune: diverse reactions to HIV test results are associated with risk behaviors. Ann Behav Med 2014; 48:371–83. 24817015 10.1007/s12160-014-9612-0 PMC4224993

[30] Feinstein BA, Johnson BA, Parsons JT, Mustanski B Reactions to testing HIV negative: measurement and associations with sexual risk behaviour among young MSM who recently tested HIV negative. AIDS Behav 2017; 21:1467–77. 27557984 10.1007/s10461-016-1525-4 PMC5528143

[31] Keaveney S, Sadlier C, O’Dea S, et al. High prevalence of asymptomatic sexually transmitted infections in HIV-infected men who have sex with men: a stimulus to improve screening. Int J STD AIDS 2014; 25:758–61. 24480850 10.1177/0956462414521165

[32] Prestage G, Brown G, Keen P Barriers to HIV testing among Australian gay men. Sex Health 2012; 9:453–8. 23380195 10.1071/SH12033

[33] Pant Pai N, Sharma J, Shivkumar S, et al. Supervised and unsupervised self-testing for HIV in high- and low-risk populations: a systematic review. PLoS Med 2013; 10:e1001414. 23565066 10.1371/journal.pmed.1001414 PMC3614510

[34] Flowers P, Knussen C, Li J, McDaid L Has testing been normalized? An analysis of changes in barriers to HIV testing among men who have sex with men between 2000 and 2010 in Scotland, UK. HIV Med 2013; 14:92–8. 22934820 10.1111/j.1468-1293.2012.01041.x PMC3561706

[35] Platteau T, Fransen K, Apers L, et al. Swab2know: an HIV-testing strategy using oral fluid samples and online communication of test results for men who have sex with men in Belgium. J Med Internet Res 2015; 17:e213. 26330138 10.2196/jmir.4384 PMC4642797

[36] Martin L, Knight V, Read PJ, McNulty A Clients’ preferred methods of obtaining sexually transmissable infection or HIV results from Sydney Sexual Health Centre. Sex Health 2013; 10:91–2. 23158695 10.1071/SH12062

[37] Flowers P, Riddell J, Park C, et al. Preparedness for use of the rapid result HIV self-test by gay men and other men who have sex with men (MSM): a mixed methods exploratory study among MSM and those involved in HIV prevention and care. HIV Med 2017; 18:245–55. 27492141 10.1111/hiv.12420 PMC5347967

[38] Spielberg F, Kurth A, Gorbach PM, Goldbaum G Moving from apprehension to action: HIV counseling and testing preferences in three at-risk populations. AIDS Educ Prev 2001; 13:524–40. 11791784 10.1521/aeap.13.6.524.21436

[39] Phillips KA, Maddala T, Johnson FR Measuring preferences for health care interventions using conjoint analysis: an application to HIV testing. Health Serv Res 2002; 37:1681–705. 12546292 10.1111/1475-6773.01115 PMC1464051

[40] Barbee LA, Dhanireddy S, Tat SA, Marrazzo JM Barriers to bacterial sexually transmitted infection testing of HIV-infected men who have sex with men engaged in HIV primary care. Sex Transm Dis 2015; 42:590–4. 26372931 10.1097/OLQ.0000000000000320 PMC4576720

